# STAT3 Suppresses Cardiomyocytes Apoptosis in CVB3-Induced Myocarditis Via Survivin

**DOI:** 10.3389/fphar.2020.613883

**Published:** 2021-01-25

**Authors:** Qiaoyu Wang, Qiongjun Zhu, Qiaofang Ye, Jiajun Wang, Qianqian Dong, Youran Chen, Minna Wang, Yu Fu, Rongzhou Wu, Tingting Wu

**Affiliations:** Children’s Heart Center, The Second Affiliated Hospital and Yuying Children’s Hospital, Wenzhou Medical University, Wenzhou, China

**Keywords:** stat3, survivin, viral myocarditis, coxsackievirus B3, apoptosis

## Abstract

**Background:** Viral myocarditis (VMC) is a common inflammatory cardiovascular disease with unclear mechanisms, which mainly affects children and adolescents. Apoptosis is the key to CVB3-induced myocarditis, and blocking this process may be beneficial to the therapy of VMC. Hence, this study aimed to explore the protective function of STAT3 on cardiomyocyte apoptosis of VMC and its underlying mechanisms.

**Methods and Results:** In this research, we confirmed that STAT3 was significantly activated in both animal and cell models of VMC. To further clarify what role did STAT3 play in VMC, AG490, an inhibitor of STAT3, was used to suppress p-STAT3. Our results demonstrated that decreased expression of p-STAT3 caused by AG490 significantly aggravated severity of VMC with elevated myocardial inflammation, deteriorative ventricular systolic function and increased mortality. It suggested that STAT3 plays a protective role in VMC. To further identify the anti-apoptosis impact that activated STAT3 made, we constructed lentivirus to regulate the expression of STAT3 in NMCs. We found that up-regulated activated STAT3 attenuated cardiomyocyte apoptosis, but down-regulated one aggravated that, which verified activated STAT3 played an anti-apoptosis role in VMC. Following that, we explored what elements are involved in the anti-apoptotic mechanism of activated STAT3 by using survivin inhibitor YM155. The result showed the anti-apoptotic effect of activated STAT3 does not work in the case of survivin inhibition.

**Conclusion:** Our findings demonstrated STAT3 by targeting survivin alleviated cardiomyocyte apoptosis in CVB3-induced myocarditis.

## Introduction

Viral myocarditis (VMC) is a kind of cardiovascular disease characterized by myocardial inflammatory infiltration. It can cause heart failure, cardio-brain syndrome, and even sudden cardiac death (Lin et al., 2016). As the pathogenesis of VMC is still unclear, there is currently no definitive diagnosis and specific treatment method (Yajima and Knowlton, 2009). Coxsackievirus B3 (CVB3), a member of the Enteroviruses genus, is the main cause of viral myocarditis (Corsten et al., 2012). It has been widely accepted that apoptosis is one of the main factors of cell damage induced by CVB3 (Seko et al., 2002). Kyto et al. observed that, after mice infected by CVB3, a large amount of apoptosis occurs in the cardiomyocytes (Kyto et al., 2004a). Henke et al. confirmed that CVB3 combined with the pro-apoptotic factor Siva to promote caspase3-dependent cell apoptosis (Henke et al., 2000). Notably, the increased rate of cardiomyocyte apoptosis in the severe viral myocarditis is closely related not only to the occurrence of fatal heart failure, but also to the transition from viral myocarditis to chronic dilated cardiomyopathy (Kyto et al., 2004b). Therefore, studying CVB3-induced apoptosis in cardiomyocytes would contribute to a better understanding of the pathogenesis of VMC and provide clues for new therapeutic treatments.

STAT3, a member of signal transducer and activator of transcription (STAT) family, is involved in transmission of various signals, including cytokines, apoptosis pathway, and so on (Haga et al., 2003; Liu et al., 2010). A great number of studies have demonstrated that activated STAT3 (phosphorylated STAT3) plays a vital role in the cardiovascular system (Negoro et al., 2000; Altara et al., 2016). Upon phosphorylation, activated STAT3 plays a role in heart remodeling of myocardial infarction and the progression of dilated cardiomyopathy, as well as in viral myocarditis (Enomoto et al., 2015). In our study, the results indicated that phosphorylated STAT3 was up-regulated in the cardiomyocytes infected by CVB3, which is in accord with the study by Yasukawa (Yasukawa et al., 2003). Herein we aimed to explore the potential role and mechanism of STAT3 in viral myocarditis.

## Methods

### Mice and Infection

All BALB/c mice were ordered from SLAC Laboratory Animal Center in China and bred at the Animal Laboratory Center of Wenzhou Medical University. All experiments were approved by the Ethics Committee of Wenzhou Medical University and in compliance with the guidelines for experimental animals. 4-week-old male BALB/c mice were randomly divided into four groups: the normal control group (NC), the AG490 treatment group (AG490), the CVB3 infection group (CVB3), the AG490 and CVB3 treatment group (AG490 + CVB3). Mice were infected with the virus on day 0. The CVB3 and AG490 + CVB3 group were injected intraperitoneally with 0.1 ml PBS containing 1 × 10^6^ PFU of purified CVB3, while the control groups were intraperitoneally injected with the sterile PBS. Besides, starting from day 1, the AG490 and AG490 + CVB3 group were intraperitoneally injected with the STAT3 inhibitor AG490 at a dose of 10 mg/kg per day. The NC and CVB3 group were intraperitoneally injected with the same amount of sterile PBS every day. The mice were sacrificed on the seventh day of the experiment. Some mouse heart specimens were fixed with 4% paraformaldehyde, and some were quickly frozen in liquid nitrogen and stored at −80°C for later use.

### Cell Culture and Infection

According to the previous protocol, newborn mice within 24 h were killed to isolate cardiomyocytes (Ehler et al., 2013). The hearts of newborn mice were cut into small pieces and transferred into the isolation medium, placed overnight on a shaker at 4°C. The isolation medium was then replaced by a digestion medium and incubated on a gentle shaker at 37°C for 30 min. Before centrifugation at 1,000 rpm for 5 mins, a 100 μm cell strainer was used to remove remaining tissue fibers. Subsequently, cells were resuspended in plating medium and then cultured in a stable environment of 37°C, 5% CO_2_, and 21% O_2_ for 40 mins. Afterward, the supernatant was transferred to a cell culture dish pre-coated with 1% gelatin (Sigma). After 1 day of cultivation, the planting liquid was replaced with maintenance liquid. After culturing in a cell culture incubator for 1 day, the cells can be used for further experiments. Under serum starvation conditions, neonatal mouse cardiomyocytes (NMC) were cultured in the medium containing CVB3 with an infection multiple of 10 to establish a viral myocarditis cell model.

### Echocardiography

Under 2% isoflurane and 0.5 L/min oxygen gas flow, mice were subjected to transthoracic echocardiography by using the Vevo 2100 imaging system (Visual Sonics, CA). Related indicators in the echocardiogram measured by supporting software include left ventricular internal diameter at end-systole (LVIDs), left ventricular internal diameter at end-diastole (LVIDd), left ventricular ejection fraction (LVEF).

### Histopathology

The mouse heart was fixed with formalin and embedded in paraffin, cut into 5 μm thick tissue sections, and stained with hematoxylin and eosin (Solarbio, China). To assess inflammation and myocardial damage, we use the myocarditis score. As mentioned in previous studies, the severity of myocarditis is assessed by the percentage of infiltrated inflammatory cells and cardiomyocyte necrosis (Yu et al., 2016).

### Western Blot

Cell lysates and heart extracts were separated by 12% SDS-PAGE and transferred to PVDF membrane. After blocked in dissolving in 5% skim milk in TBS for 2 h, incubate in p-STAT3, STAT3, survivin, Cleaved Caspase-3, Caspase-3, Bax (Cell Signaling Technology) and Bcl-2(Santa Cruz) antibody, and overnight at 4°C. After washed with TBST, the membrane was placed in a box containing goat anti-rabbit IgG for 2 h. After combined with ECL developer, the membrane was developed under the Bio-Rad gel imaging system.

### Real-Time Polymerase Chain Reaction

The RNA extracted by the TRIZOL method was reverse transcribed into cDNA (TransGen Biotech, China). Quantitative Real-time PCR was used to detect the expression of STAT3 and survivin. All primers were ordered from Sangon Biotech Co., Ltd. (Shanghai, China). The following primers were used: survivin, the forward primer 5′- GAG​GCT​GGC​TTC​ATC​CAC​TG-3′, and reverse primer 5′- CTT​TTT​GCT​TGT​TGT​TGG​TCT​CC-3′; GAPDH, the forward primer 5′-AGG​TCG​GTG​TGA​ACG​GAT​TTG-3′ and reverse primer 5′-TGT​AGA​CCA​TGT​AGT​TGA​GGT​CA-3′; STAT3, the forward primer 5′-CAA​TAC​CAT​TGA​CCT​GCC​GAT-3′ and 5′-GAG​CGA​CTC​AAA​CTG​CCC​T-3′.

### TUNEL Assay

Apoptosis was evaluated by TUNEL Assay (Roche). The cells or tissues were fixed with 4% paraformaldehyde at room temperature for 20 mins and placed in 0.1% Triton X-100 solution on ice for 10 mins. The samples were covered with a 30 μl TUNEL reaction solution and incubated in the humid and dark environment at 37°C for an hour. Then samples were reacted with DAPI fluorescent dye solution for 5 mins. A fluorescence microscope captured the images.

### Assessment of Cardiomyocytes Injury

LDH-Cytotoxicity Assay Kit (Beyotime) was applied to assess the severity of cardiomyocytes damage. 120 μL/well supernatant of each sample was transferred to a 96-well plate and mixed with 60 µL working solution. After incubated at room temperature for 30 mins in the dark, the absorbance was measured at 490 nm.

### Statistical Analysis

When using GraphPad Prism seven software for analysis, results were expressed as mean ± standard error. To analyze the statistical significance between multiple groups, one-way analysis of variance was used. To analyze multiple treatment experiments, a two-way analysis of variance was used. The log rank test was used to analyze the survival rate of different groups. The *p*-value < 0.05 was considered significant.

## Results

### STAT3 Was Activated in the CVB3-Induced Viral Myocarditis

**FIGURE 1:**
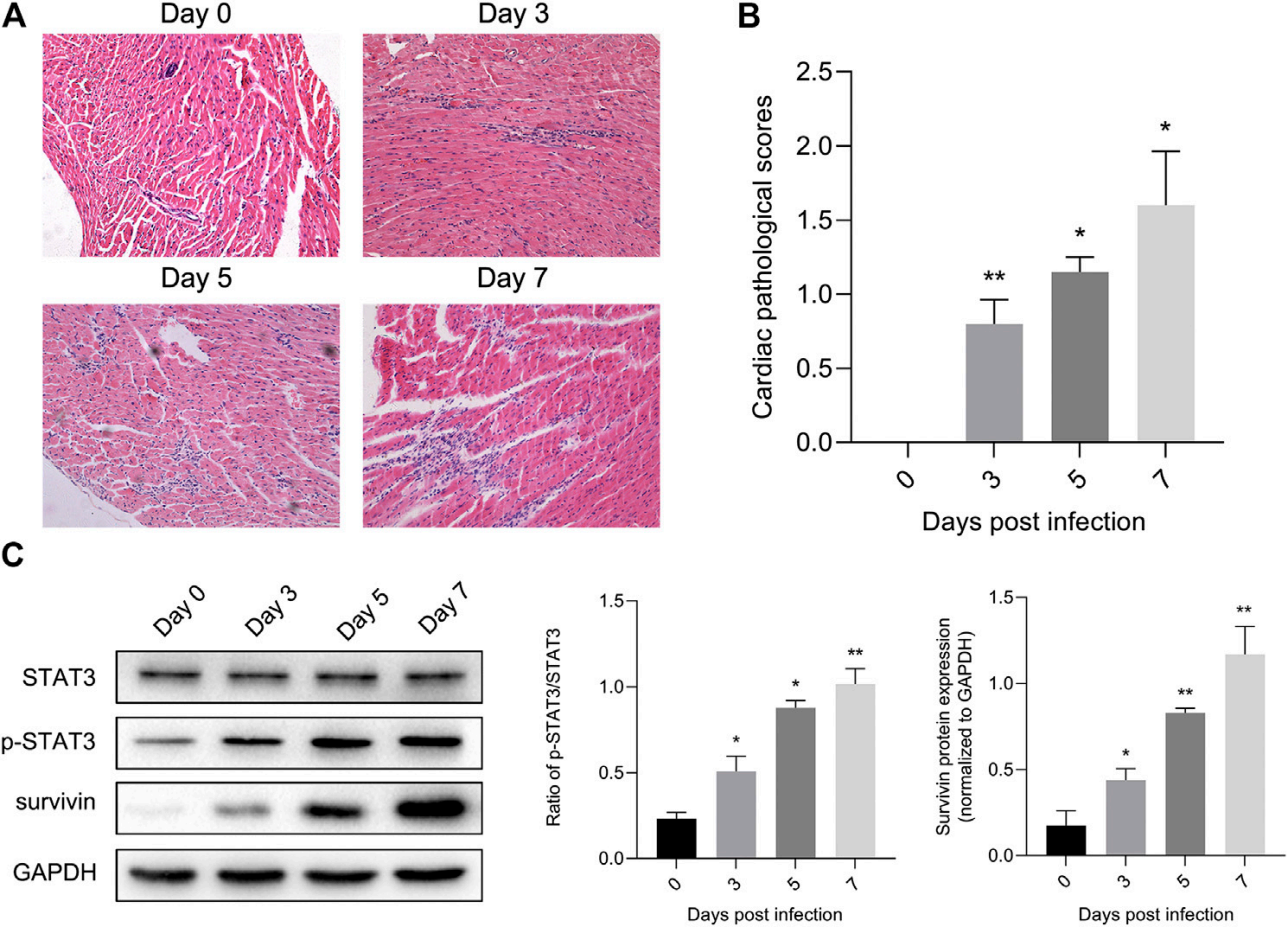
STAT3 was activated in viral myocarditis animal model. Mice were injected intraperitoneally with CVB3 and hearts were collected at designated time points **(A)** Cardiac damage was quantified on hematoxylin and eosin staining (*n* = 4, 200×) **(B)** The cardiac pathological scores in different groups (*n* = 4) **(C)** The related proteins were measured by western blot (*n* = 4). *p < 0.05, **p < 0.01 vs control group (NC).

Previous studies have illustrated the protective effect of STAT3 on autoimmunity (Camporeale et al., 2013) and CVB3-induced myocarditis (Yajima et al., 2006). To validate this finding, we extracted mouse heart tissue infected with CVB3 to detect STAT3 and phosphorylated STAT3 (p-STAT3) protein levels, as well as to perform inflammation histopathological examination. Myocarditis began to appear 3 days post CVB3 infection, and inflammation of the myocardium gradually increased over time (Figures 1A,B). In addition, the expression of p-STAT3 remained at a low level in the normal mice, but increased along with the prolongation of CVB3-infected time (Figure 1C).

**FIGURE 2:**
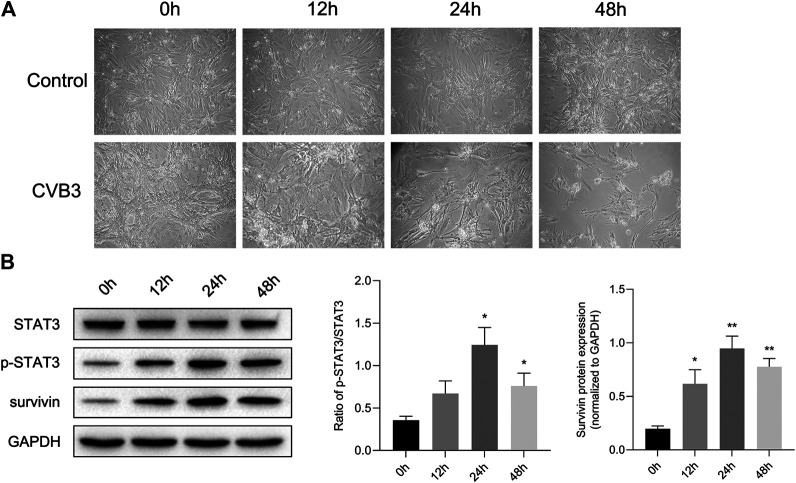
STAT3 was activated in viral myocarditis cell model. NMCs were infected with CVB3 and the proteins were collected at different time point **(A)** The morphological changes of NMC at 0, 12, 24, and 48 h after CVB3 infection (200×) **(B)** The related proteins were measured by western blot (*n* = 4). *p < 0.05, **p < 0.01 vs control group (NC).

At the same time, we constructed a viral myocarditis model of NMCs. There were no significant changes in the morphology and pulsation of cardiomyocytes compared with the control group at 12 h post infection (pi); Then some cardiomyocytes showed a wire-drawing phenomenon with the beating frequency slowing down and even irregular meanwhile at 24 h pi; Finally the cardiomyocytes gradually shrank and became round, and even detached and dissolved at 48 h pi (Figure 2A). What’s more, western blotting assay showed that the expression of p-STAT3 reached a peak after infected for 24 h, and then appeared to drop (Figure 2B). This further confirmed that CVB3 could stimulate the expression of p-STAT3, and suggested that the p-STAT3 might be related to the pathogenesis of CVB3-induced myocarditis.

### Protective Effect of Activated STAT3 in the Viral Myocarditis

**FIGURE 3:**
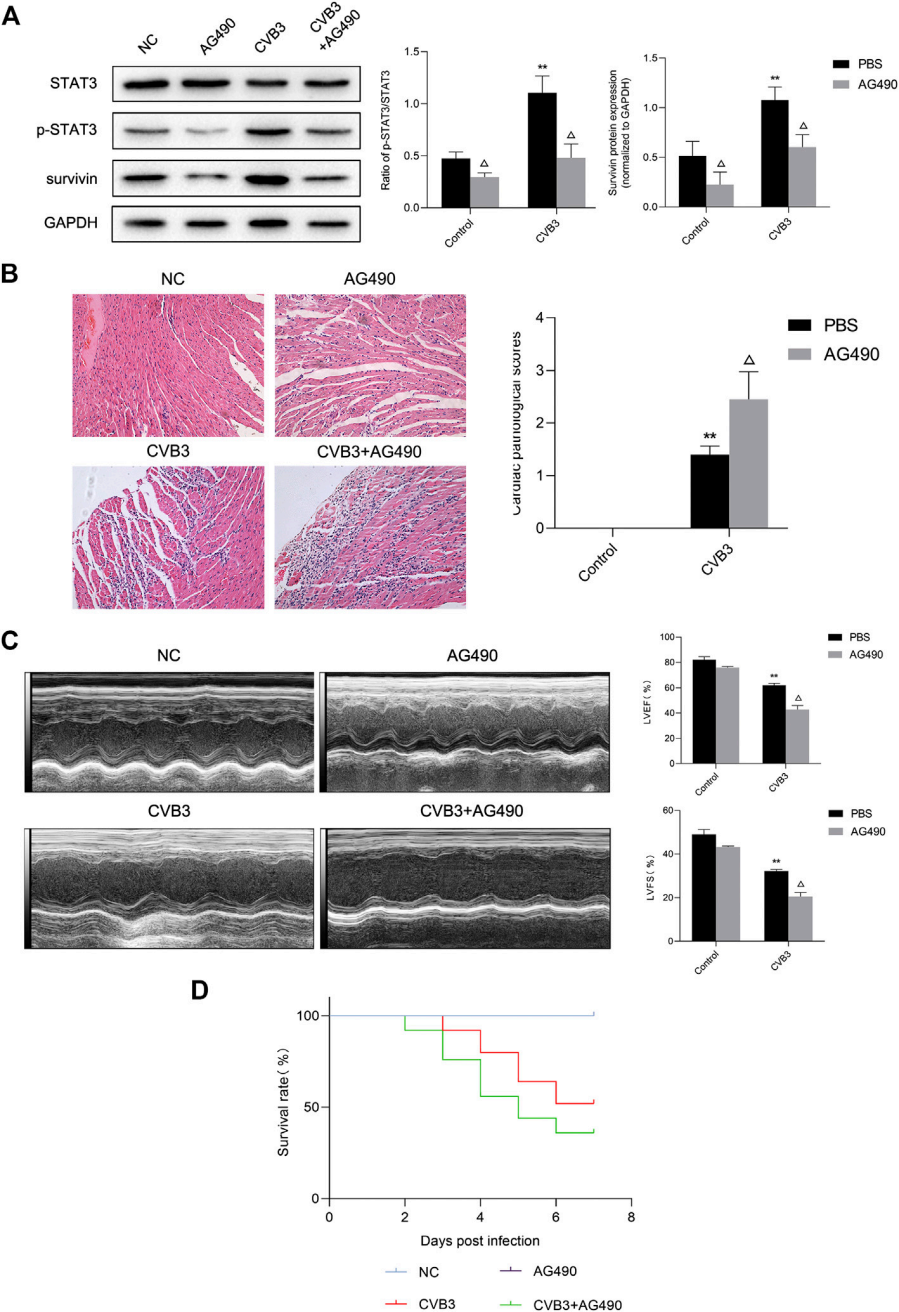
Protective effect of activated STAT3 in the viral myocarditis **(A)** The related proteins were measured by western blot (*n* = 4). **p < 0.01 vs control groups (NC and AG490 group), ^△^ *p* < 0.05 vs CVB3 groups (CVB3 and AG490 + CVB3 group) **(B)** HE staining of myocardium (*n* = 4,200×) and cardiac pathological scores (*n* = 4) **p < 0.01vs control groups (NC and AG490 group), ^△^ *p* < 0.05 vs CVB3 groups (CVB3 and AG490 + CVB3 group) **(C)** M-mode echocardiogram images and analysis of LVEF and LVFS in different groups (*n* = 3). **p < 0.01vs control groups (NC and AG490 group), ^△^ *p* < 0.05 vs CVB3 groups (CVB3 and AG490 + CVB3 group) **(D)** Survival analysis of viral myocarditis mice. Survival proportions at day 7 were 100% for the NC group and AG490 group, 52% for the CVB3 group, 36% for the CVB3+AG490 group. **p < 0.01 vs control groups (NC and AG490 group), ^△^ *p* < 0.05 vs CVB3 groups (CVB3 and AG490 + CVB3 group).

Next, we tried to clarify what role STAT3 played in viral myocarditis. For this purpose, we injected STAT3 inhibitor AG490 into the abdominal cavity of mice with viral myocarditis. We confirmed that AG490 eliminated the upregulation of p-STAT3 induced by CVB3 through western blot (Figure 3A). Then we conducted HE staining of extracted mouse heart tissue at 7 days pi to assess myocarditis severity. Multifocal necrosis and inflammatory infiltration appeared to the myocardium of myocarditis mice, which were aggravated when treated with AG490 (Figure 3B).

Echocardiography was used to evaluate murine cardiac function by calculating left ventricular fractional shortening (LVFS) and left ventricular ejection fraction (LVEF). After CVB3 inoculation, the LVFS and LVEF of mice significantly decreased. Compared with CVB3-infected mice, the LVFS and LVEF of AG490-treated myocarditis mice was significantly reduced by 36.38% and 31.01% respectively, indicating that ventricular function worsened after AG490 treatment (Figure 3C). What’ more, only 36% of AG490-treated myocarditis mice survived until Day 7 (Figure 3D). These data illustrated that down-regulation of activated STAT3 can significantly aggravate the severity of CVB3-induced myocarditis.

### Anti-Apoptotic Effect of Activated STAT3 in the Viral Myocarditis

**FIGURE 4:**
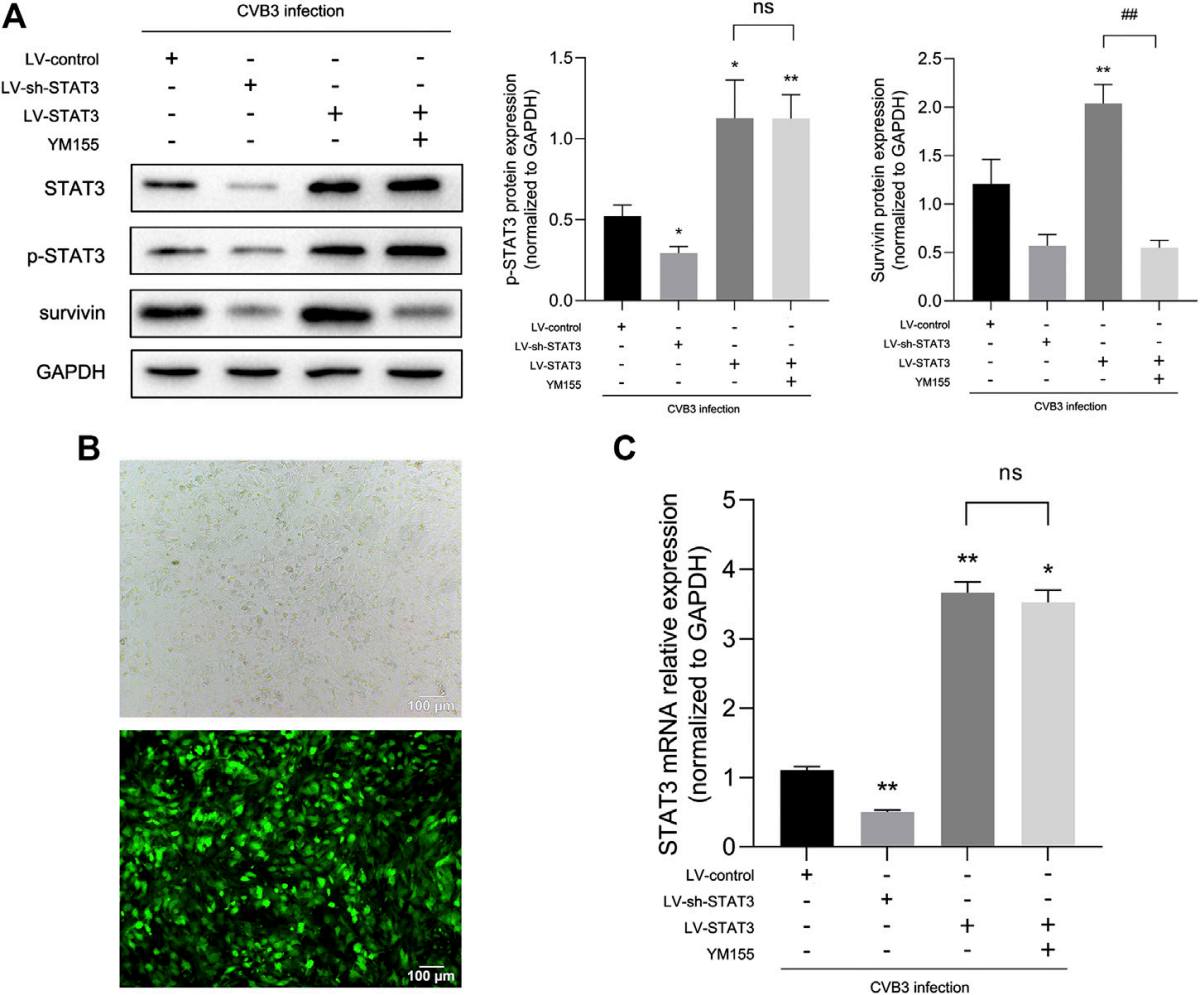
Regulatory effects of lentiviral tools **(A)** The related proteins were measured by western blot (*n* = 4). **(B)** Expression of GFP in NMCs at 72 h after lentivirus transfection (100×). **(C)** The relative mRNA expression of STAT3 compared to GAPDH (*n* = 3). *p < 0.05, **p < 0.01vs Lv-control group, ns no significance, ^#^ *p* < 0.05, ^##^ *p* < 0.01 vs Lv-STAT3 group.

To further determine the effect of STAT3 in viral myocarditis, we constructed Lv-STAT3 and Lv-sh-STAT3 to regulate the level of p-STAT3 in NMCs. As demonstrated in Figure 4B, the lentivirus has high transfection efficiency with hardly affection to the vitality of NMCs. Meanwhile, compared with the Lv-control group, CVB3-infected cardiomyocytes transfected with Lv-STAT3 showed high expression of STAT3 mRNA, while Lv-sh-STAT3 down-regulated the mRNA level of STAT3 (Figure 4C). In addition, we detected the STAT3 and p-STAT3 protein levels of all groups (Figure 4A), which were aligned with the trend of mRNA expression, confirming the lentiviral vector can effectively regulate the expression of p-STAT3.

**FIGURE 5:**
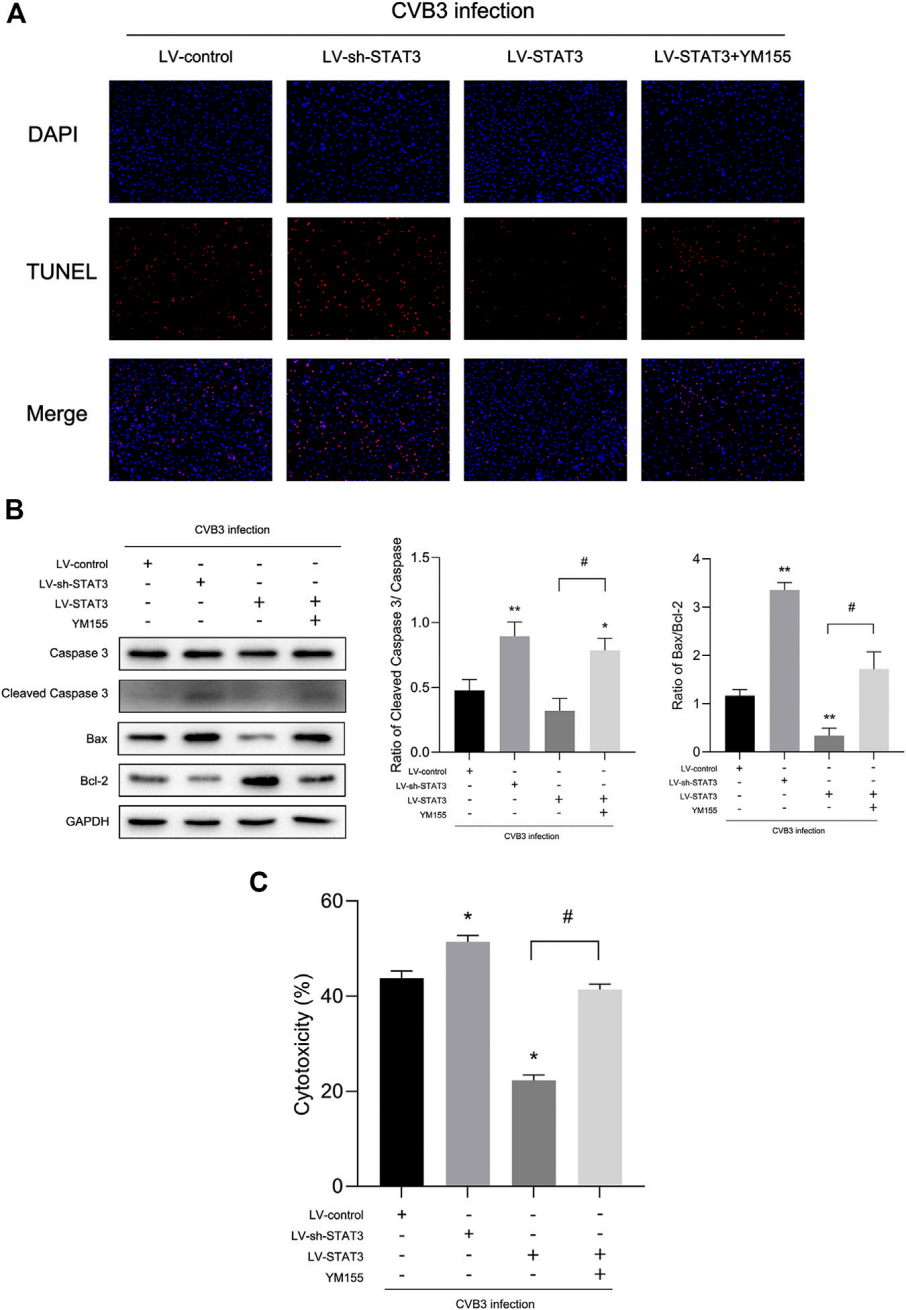
Activated STAT3 inhibited CVB3-induced cardiomyocyte apoptosis via survivin **(A)** Photomicrographs (100×) of apoptotic cells. **(B)** The expression of Bax and Cleaved Caspase-3 was measured by western blot (*n* = 4). **(C)** The cytotoxicity tested by LDH assay kit in different groups (*n* = 4). *p < 0.05, **p < 0.01 vs Lv-control group, ^#^ *p* < 0.05 vs Lv-STAT3 group.

**FIGURE 6:**
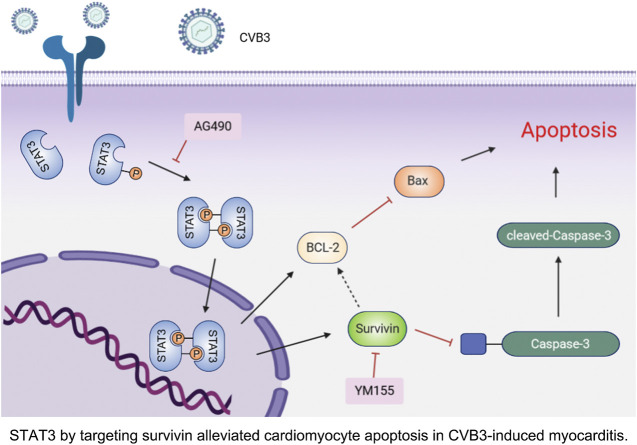
STAT3 by targeting survivin alleviated cardiomyocyte apoptosis in CVB3-induced myocarditis.

Since activated STAT3 may play a protective role in viral myocarditis, the effect of activated STAT3 on cardiomyocyte damage caused by CVB3 infection was evaluated through LDH assay. When treated with the Lv-sh-STAT3, the LDH of the CVB3-infected NMCs was sharply increased, while the release of LDH in the Lv-STAT3 group decreased (Figure 5C). Given that cell apoptosis is one of the mechanisms in viral myocarditis and one of the important manifestations in myocardial damage (Hasslacher et al., 2011), we tested the impact of activated STAT3 in the regulation of cardiomyocyte apoptosis. We found a higher level of the apoptosis-related protein Cleaved Caspase-3 and Bax after NMCs infected with CVB3 for 24 h (Figure 5B). It was worth mentioning that Lv-STAT3 downregulated Cleaved Caspase-3 and Bax protein, while Lv-sh-STAT3 markedly upregulated their expression. Meanwhile, in the TUNEL assay, it was further revealed that Lv-STAT3 attenuated cardiomyocyte apoptosis caused by CVB3, while Lv-sh-STAT3 aggravated those (Figure 5A). These results indicated that activated STAT3 has an anti-apoptotic effect on NMCs infected with CVB3.

### Activated STAT3 Inhibited CVB3-Induced Cardiomyocyte Apoptosis Via Survivin

Survivin, a member of the inhibitor-of-apoptosis protein (IAP) family, inhibits caspase activation, thereby preventing cell apoptosis among some cardiovascular diseases (Santini et al., 2004; Yang et al., 2015). Our previous study (Li et al., 2019) also found that highly-expressed survivin exerted myocardial protection through negative regulation of apoptosis in myocarditis. In that way, whether or not does survivin involve in the anti-apoptosis from activated STAT3 on myocardium? Recent reports suggest a possible collaborative relationship between STAT3 and Survivin (Martinez-Garcia et al., 2019; Rajina et al., 2020). Consistent with this conclusion, once the CVB3-infected NMCs were transfected with lentivirus to regulate the expression of STAT3, highly corresponding regulation appeared to survivin as well (Figures 1, 2). This synchronized change suggests that survivin may be a downstream target protein of STAT3 in viral myocarditis.

To further clarify whether STAT3 plays an anti-apoptotic role through survivin, we introduced a survivin inhibitor, YM155, which is a novel, small, imidazolium-based compound that suppresses survivin via transcriptional inhibition of the survivin gene promoter. When we added YM155 and Lv-STAT3 simultaneously, the upregulation of survivin induced by Lv-STAT3 was reversed (Figure 4A). Whereafter, as demonstrated by TUNEL assay (Figure 5A), compared with the Lv-STAT3 group, CVB3-induced apoptosis of cardiomyocytes increased again when treated with both YM155 and Lv-STAT3. It suggested that the anti-apoptotic effect of STAT3 activation does not work in the case of survivin inhibition. Same results were obtained in western blot (Figure 5B) and cytotoxicity experiment (Figure 5C), that is the upregulation of apoptosis-related protein and LDH release in the group which treated with both YM155 and Lv-STAT3 compared with the Lv-STAT3 group. Overall, these findings demonstrated that activated STAT3 achieved protective action in CVB3-induced myocarditis through survivin.

## Discussion

Viral myocarditis is an acute inflammatory disease of the heart, and is currently a leading cause of sudden death in children and young adults (Qiu et al., 2017). Nevertheless, the specific mechanism of VMC remains obscure. Exploring the mechanism and identifying an early and effective treatment have important clinical significance for improving the quality of life of patients with viral myocarditis and for reducing mortality. STAT3 is strictly controlled under physiological conditions and is abnormally active under pathological conditions such as inflammation and ischemia (Yu et al., 2009; Haapaniemi et al., 2015). In the present study, we observed that p-STAT3 levels were low under normal conditions. After CVB3 infects BALB/c mice, we clearly observed up-regulation of p-STAT3 protein. Protein expression of p-STAT3 was time-dependent and was coincident with the pathological changes of myocardium (Figure 1). This phenomenon was also demonstrated in the cell model of viral myocarditis. Level of p-STAT3 reached a peak at 24 h. Subsequently, the protein p-STAT3 began to decline. Simultaneously, the infected NMCs gradually shrank and became round, and even detached and dissolved (Figure 2). These findings suggest that STAT3 was activated in the CVB3-induced viral myocarditis, and may be related to pathophysiological process of VMC.

Previous studies found that STAT3 protected the heart, and mutations in STAT3 made the heart more vulnerable (Tsai et al., 2008; Su et al., 2017; Wang et al., 2018). To confirm the function of STAT3 activation in viral myocarditis, the STAT3 inhibitor AG490 was applied to the animal model of VMC. We found that down-regulation of p-STAT3 mediated by AG490 exacerbated CVB3-induced cardiac injury, manifested by increased myocardial inflammation, deterioration of left ventricular function, and decreased survival rate (Figure 3). These results demonstrated that activated STAT3 plays a protective role in VMC to a certain extent.

Apoptosis is programmed cell death, primarily characterized by the formation of apoptotic bodies. To initiate apoptosis, cells activate caspase-3 through exogenous death receptors and endogenous mitochondrial pathways. Apoptosis maintains the body's physiological homeostasis and participates in many pathophysiological processes, including ischemia, hypoxic injury, and viral pathogenesis (Li et al., 2014; Shim et al., 2017; Zhou et al., 2017). A number of studies found apoptosis was related to VMC-induced heart damage and leads to myocardial remodeling (Fuse et al., 2000; Kang and Izumo, 2000; Abbate et al., 2009). STAT3 performs a vital function in controlling cell fate, including cell growth, proliferation, and apoptosis (Inghirami et al., 2005; Yu et al., 2009). Qaed et al. reported that phosphocreatine protected the heart from apoptosis by upregulating levels of p-STAT3 in mice with diabetic cardiomyopathy (Qaed et al., 2019). Son et al. found that siRNA-STAT3 treatment increased cell apoptosis (Son et al., 2017). To clarify the relationship between STAT3 and apoptosis in the VMC cell model, western blot was used to measure the expression of these proteins. After the transfection of Lv-STAT3, levels of p-STAT3 and STAT3 protein increased significantly, while levels of Cleaved Caspase-3 and Bax protein were down-regulated. By contrast, higher levels of Cleaved Caspase-3 and Bax were found in the Lv-sh-STAT3 group of the infected NMCs, compared with the CVB3-infected group. Of note, at the same time, apoptosis of cardiomyocytes was remarkably inhibited by Lv-STAT3 as shown in the TUNEL assay (Figure 5). Taken together, these findings suggest that activated STAT3 exerts anti-apoptotic action in CVB3-infected NMCs.

We next explored those elements that are involved in the anti-apoptotic mechanism of STAT3 activation. As a well-known inhibitor of apoptosis, survivin blocks the activation of caspase three and exerts an effective anti-apoptotic function in cell lines and animal models (Li, 2003; Bertero et al., 2013; Lossi et al., 2016). Although early research on survivin mainly focused on proliferating cells, several lines of evidence suggest that inflammation and hypoxia also stimulate survivin expression in quiescent cells (Sah et al., 2006; Yang et al., 2015). Previous studies found that there was a high expression levels of survivin in the myocardial tissue of patients with myocardial infarction (Santini et al., 2004). In a study of myocardial ischemia, it was also evident that the expression of survivin protein in cardiomyocytes increased significantly after ischemia-reperfusion injury (Yang et al., 2015). In our previous studies, we noticed that survivin overexpressed in viral myocarditis and the up-regulation of survivin negatively correlated with the expression of Cleaved Caspase-3, suggesting that survivin may protect cardiomyocytes from CVB3- induced apoptosis (Li et al., 2019; Wu et al., 2020; Yu et al., 2015). Martínez-García et al. reported that T21 inhibited STAT3 phosphorylation in lung cancer, which reducing the gene expression of survivin (Martinez-Garcia et al., 2019). Interestingly, in the present study, we obtained novel findings to the effect that the expression of survivin was synchronized with that of STAT3 in a time-dependent manner in the context of viral myocarditis (Figures 1, 2). It is worth mentioning that changes in survivin protein expression were highly consistent with those of p-STAT3, as shown in experiments where we regulated the expression of STAT3 in cardiomyocytes using lentivirus (Figure 4). These findings suggested that survivin might be a downstream functional target gene of STAT3 in VMC, and this has never been mentioned in the previous literature. Nevertheless, whether STAT3 inhibits apoptosis in viral myocarditis through survivin requires more in-depth research. Accordingly, survivin inhibitor YM155 was used to further evaluate the function of activated STAT3. Compared with the Lv-STAT3 group, apoptosis-related protein and LDH release were re-elevated when treated with both YM155 and Lv-STAT3 (Figure 5), which mean that even if STAT3 is overexpressed, it cannot effectively exert its anti-apoptotic effect after blocking the expression of survivin. From this, we can determine that survivin serves as a mediator of STAT3 anti-apoptosis in CVB3-induced myocarditis.

In summary, our study demonstrated that activated STAT3 protects cardiomyocytes from CVB3-induced apoptosis *in vivo* and *in vitro*. More importantly, we confirmed for the first time that this anti-apoptotic function depends on the up-regulation of survivin (Figure 6). These novel observations provide several new insights into the underlying pathogenesis of viral myocarditis, which may be beneficial to the development of effective therapies.

## Data Availability

The raw data supporting the conclusions of this article will be made available by the authors, without undue reservation.
