# Supplementary material for: STAT3 Suppresses Cardiomyocytes Apoptosis in CVB3-Induced Myocarditis Via Survivin
Source: Front Pharmacol. 2021 Jan 25;11:613883. doi: 10.3389/fphar.2020.613883 (PMC7919905; doi:10.3389/fphar.2020.613883)
Supplement: Supplementary file 1 [file Image1.pdf]

Original image of the article

Figure 1

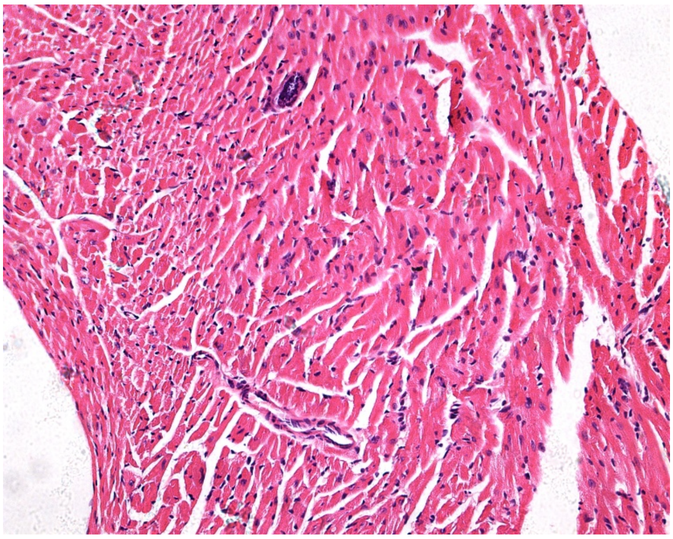

Figure 1A. Cardiac damage was quantified on hematoxylin and eosin staining (Day 0 group)

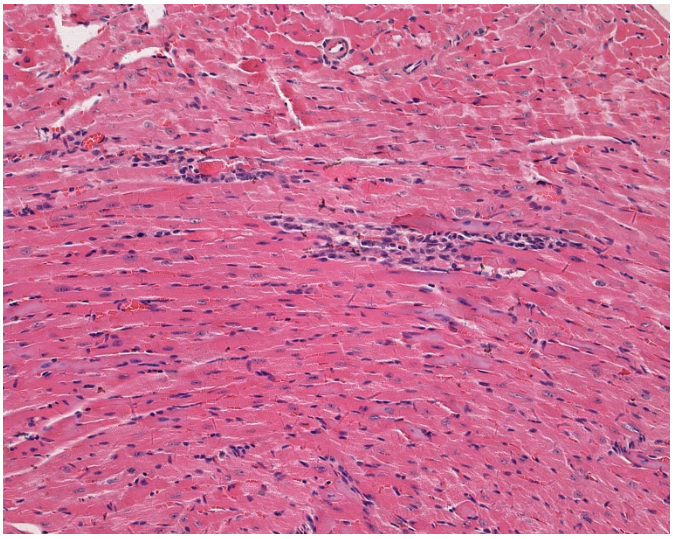

Figure 1A. Cardiac damage was quantified on hematoxylin and eosin staining (Day 3 group)

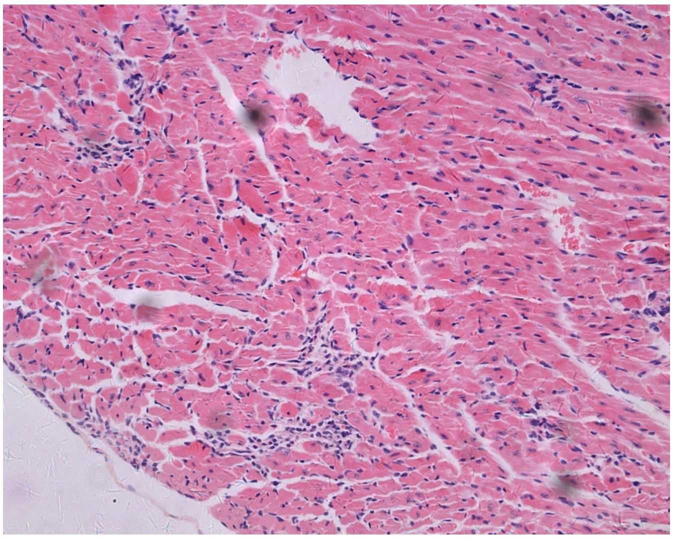

Figure 1A. Cardiac damage was quantified on hematoxylin and eosin staining (Day 5 group)

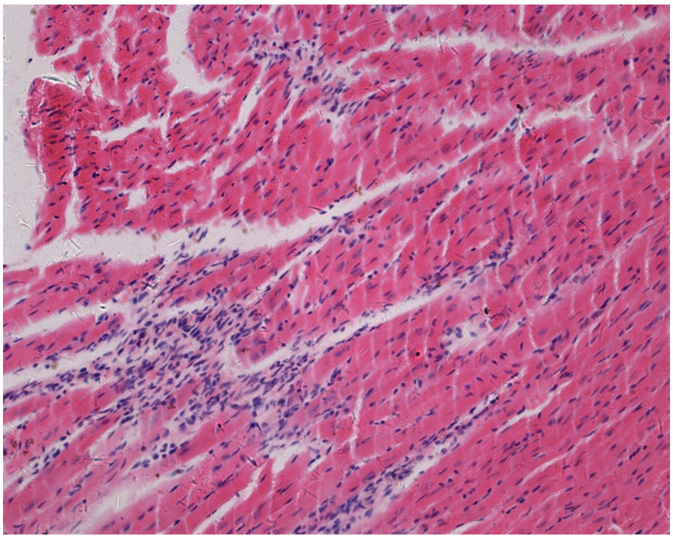

Figure 1A. Cardiac damage was quantified on hematoxylin and eosin staining (Day 7 group)

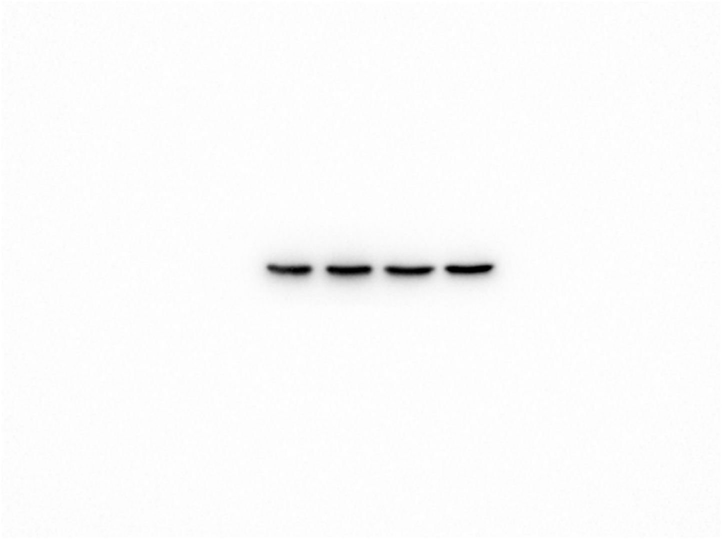

Figure 1C. GADPH protein expression

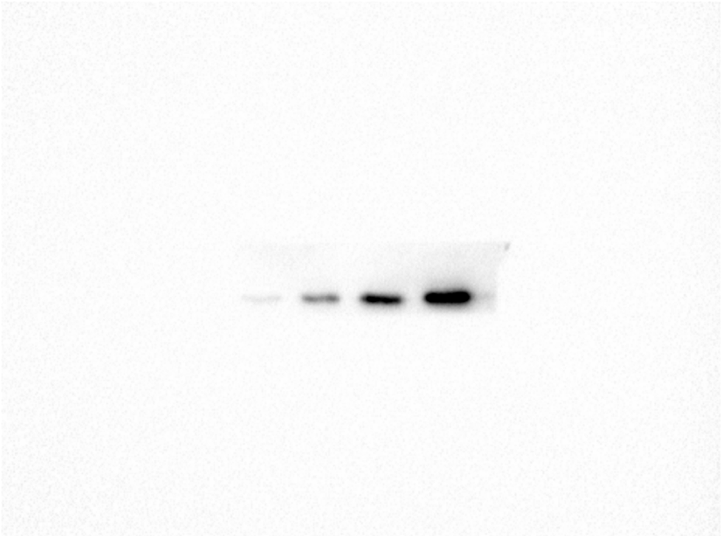

Figure 1C. survivin protein expression

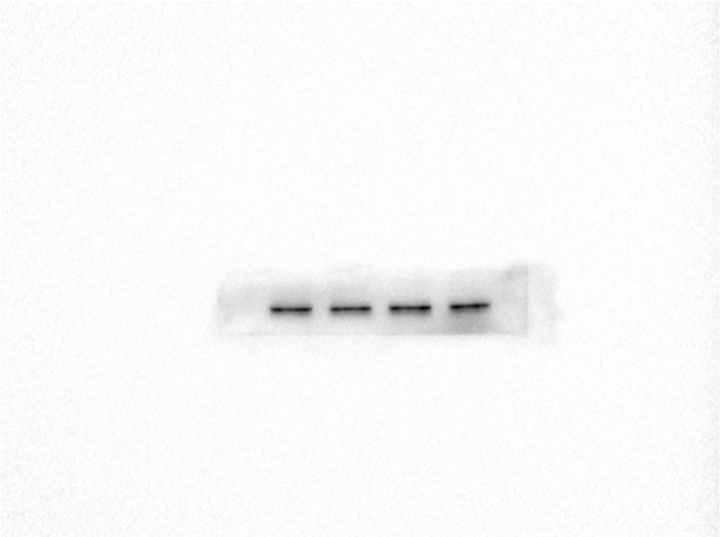

Figure 1C. STAT3 protein expression

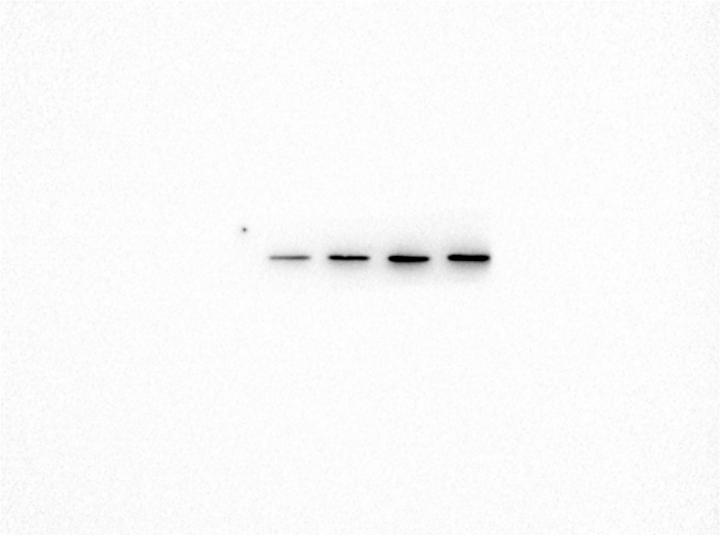

Figure 1C. p-STAT3 protein expression
